# Identification and characterization of a POU transcription factor in the cotton bollworm, *Helicoverpa armigera*

**DOI:** 10.1186/1471-2199-10-25

**Published:** 2009-03-25

**Authors:** Tian-Yi Zhang, Wei-Hua Xu

**Affiliations:** 1 State Key Laboratory of Biocontrol, School of Life Sciences, Sun Yat-Sen (Zhongshan) University, Guangzhou 510275, PR China; 2 Current address : Department of Ophthalmology, Harvard Medical School and Massachusetts Eye and Ear Infirmary, Boston, Massachusettes, USA

## Abstract

**Background:**

The POU family genes containing the POU domain are common in vertebrates and invertebrates and play critical roles in cell-type-specific gene expression and cell fate determination.

**Results:**

Har-POU, a new member of the POU gene family, was cloned from the suboesophageal ganglion of *Helicoverpa armigera *(Har), and its potential functions in the development of the central nervous system (CNS) were analyzed. Southern blot analysis suggests that a single copy of this gene is present in the *H. armigera *haploid genome. Har-POU mRNA is distributed widely in various tissues and expressed highly in the CNS, salivary gland, and trachea. *In vitro*-translated Har-POU specifically bound canonical octamer motifs on the promoter of diapause hormone and pheromone biosynthesis activating neuropeptide (DH-PBAN) gene in *H. armigera*. Expression of the Har-POU gene is markedly higher in the CNS of nondiapause-destined pupae than in diapause-destined pupae. Expression of the Har-POU gene in diapausing pupae was upregulated quickly by injection of ecdysone.

**Conclusion:**

Har-POU may respond to ecdysone and bind to the promoter of DH-PBAN gene to regulate pupal development in *H. armigera*.

## Background

The development of many insects is regulated by environmental signals such as photoperiod, temperature, humidity, and nutrients. The central nervous system (CNS) of insects accepts these environmental stimuli and transduces them into endogenous chemical messengers (neuropeptides or hormones) in the neuroendocrine organs [1]. The neuropeptides and hormones induce insect developmental arrest at a certain stage: embryonic, larval, pupal, or adult. The programmed arrest of development is called diapause. In *Bombyx mori *(Bom), embryonic diapause is caused by a neuropeptide diapause hormone (DH), which is secreted from the suboesophageal ganglion (SG) and acts on the developing ovaries of the pharate adult to induce the laying of diapause eggs in the next generation [2]. Interestingly, neuropeptide pheromone biosynthesis activating peptide (PBAN), which can stimulate the pheromone gland of female adults to secrete sex pheromone to attract male adults for mating in Lepidoptera, is also encoded by DH cDNA [3]. Thus, the DH cDNA and gene also are referred as the DH-PBAN cDNA and DH-PBAN gene, respectively [4]. DH-PBAN cDNAs have been cloned from a number of Lepidoptera species.

The DH-PBAN gene was first cloned in *B. mori *[4]. The transcription factor POU-M2, a member of the POU family of genes, interacts specifically with the promoter of the Bom-DH-PBAN gene and regulates its transcription [5]. Initially, the transcription factors Pit, Oct, and Unc (POU) were found to possess a conserved DNA binding region of approximate 160 amino acids, and the conserved DNA binding region was then designated as the POU domain [6]. The POU domain consists of two subdomains, the POU-specific domain and a homeobox domain. The POU family genes containing the POU domain are common in vertebrates and invertebrates and play critical roles in cell-type-specific gene expression and cell fate determination [7]. Based on the variations of POU-homeobox domains, they are divided into six classes (POU-I~-VI), with POU-III the largest class [8,9].

One of the main functions of POU-III members in mammals is to regulate the development of the CNS and neuroendocrine system and the expression of some neurohormones [10]. In insects, POU-III members have been cloned from *Drosophila melanogaster *(*drifter*) and *Bombyx mori *(POU-M1/M2). *drifter *from *D. melanogaster *was first identified as a neuron-specific regulator binding the C element of the dopa decarboxylase gene [11]. Later, *drifter *was found to be involved in multiple important developmental events: differentiation and migration of tracheal cells and neurons [12-14], cell fate determination of *Drosophila *imaginal discs [15], and neuronal lineage and wiring [16-19]. POU-M1 from *B. mori *was cloned from the silk gland and was found to bind the SC element of the sericin-1 gene and regulate its transcription [20]. POU-M2 is an isoform of POU-M1 and regulates expression of the *B. mori *DH-PBAN gene, an important neuropeptide related to development [5].

Recently, a second DH-PBAN gene from the pupal diapause species *Helicoverpa armigera *(Har) (Lepidoptera: Noctuidae) was cloned and showed a potential binding site for the POU in the promoter sequences [21]. Although the DH-PBAN gene is involved in controlling egg diapause in *B. mori *and pupal diapause in *H. armigera*, the respective mechanisms might be different, and the two temporal patterns are significantly different. The DH-PBAN mRNA content in *B. mori *pupae destined to lay diapause eggs is 2.7 times higher than that of pupae destined to lay nondiapause eggs [22]. The DH-PBAN mRNA content in diapause-type pupae of *H. armigera *is significantly lower than that of nondiapause-type pupae [23]. Therefore, we want to clarify whether the transcription factor POU is involved in regulating DH-PBAN expression to control pupal diapause in *H. armigera*.

Here we report the cloning and characterization of a cDNA encoding POU in *H. armigera *(Har-POU) as well as analysis of its genome copy, tissue distribution, and DNA binding activity. Furthermore, we show that developmental expression of the Har-POU gene is much higher in the CNS of nondiapause pupae than that of diapause-type pupae. Thus, expression of the Har-DH-PBAN gene is closely correlated with the response to the Har-POU transcription factor.

## Results

### Isolation and sequence analysis of Har-POU

**Figure 1:**
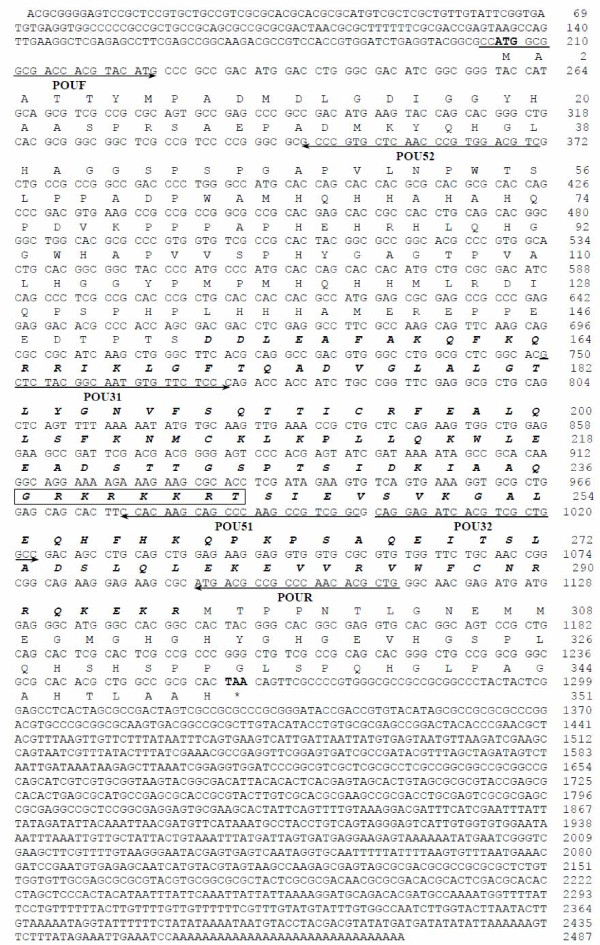
**Nucleotide and amino acid sequences of a cDNA encoding Har-POU**. The potential nuclear localization signal sequence is shown in the rectangle, and the POU-specific and homeobox domains are shown in bold italics. Arrows below the nucleotide sequences represent the position of the different synthetic primers used in PCR. Degenerate primers are POUF (5'-CCATGGCGGCGAC(C/G)AC(C/G)TA(C/T)ATG-3') and POUR (5'-CAGCGTGTTGGG(C/T)GG(C/T)GTCAT-3'). This sequence has been submitted to GenBank (accession number: AY513764).

By using degenerate primers POUF and POUR based on the nucleotide sequences conserved between *B. mori *and *D. melanogaster*, PCR amplification yielded a product of approximately 900 bp. The amino acid sequence encoded by this cDNA fragment shows 96.7% and 54.5% similarity with the corresponding regions of POU from *B. mori *and *D. melanogaster*, respectively. To obtain the full-length POU cDNA, 5'- and 3'-RACE were performed with specific primers based on the sequence of the 900-bp cDNA fragment. Approximately 370 bp at 5'-end and 1500 bp at 3'-end were amplified by PCR, and the two fragments were subcloned into vectors and sequenced. The full-length cDNA is 2455 bp, including a 204-bp 5' untranslated region, 1056-bp open-reading frame, and a 1195-bp 3' untranslated region (Fig. 1). This sequence has been submitted to GenBank (accession number: AY513764).

**Figure 2:**
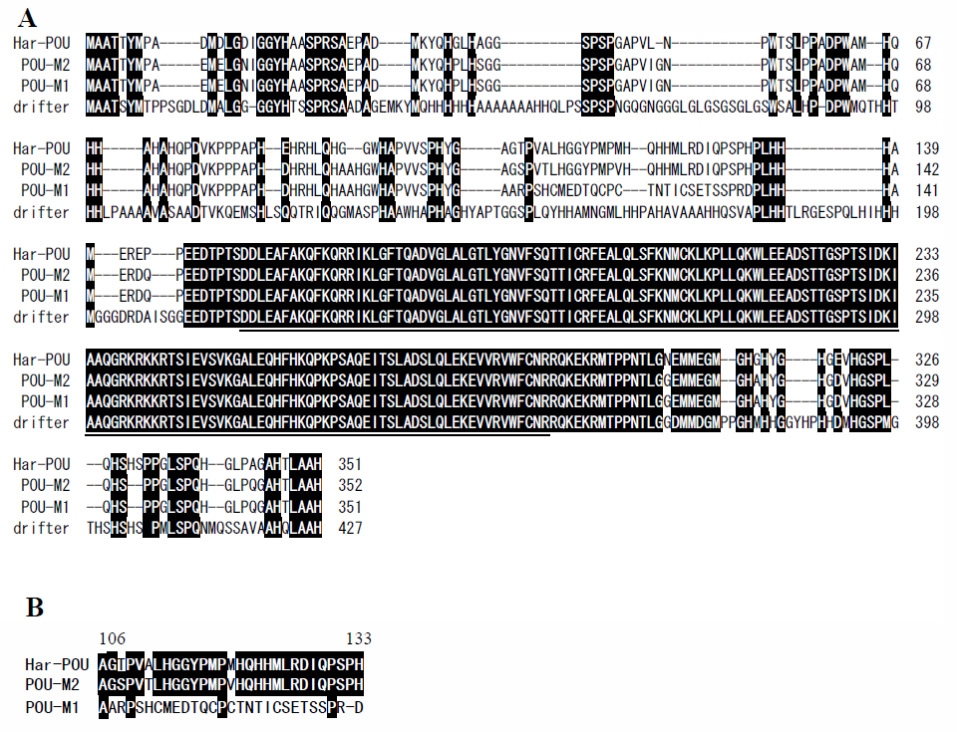
**Sequence alignment of Har-POU with POU-M1/-M2 from *B. mori *and Drifter from *D. melanogaster***. (A) The POU-specific and homeobox domains are underlined. Identical residues are shaded in black. The numbers on the right indicate the amino acids of each protein. (B) Amino acid sequence similarities of 28 amino acids from residue 106 to residue 133 of POUs. The highly conserved residues (≥67%) are shaded in black.

The Har-POU cDNA encodes 351 amino acids containing a conserved class POU-III-specific and homeobox domain located on amino acids 153–296. Alignment shows that the POU domain of Har-POU is the same as *drifter *in *Drosophila *and POU-M1/M2 in *B. mori *(Fig. 2A), and only two amino acids differ from that of APH-1, another arthropod POU-III gene of *Artemia franciscana *[24]. The POU domain also has high similarity with other POU-III genes in *C. elegans *and mammals (data not shown). The similarity at the N-terminal of Har-POU is 78.1%, 93.5%, and 29.0%; and at C-terminal is 92.5%, 92.5%, and 61.5% to POU-M1, POU-M2, and *drifter*, respectively (Fig. 2B).

### Har-POU copy number in H. armigera haploid genome

**Figure 3:**
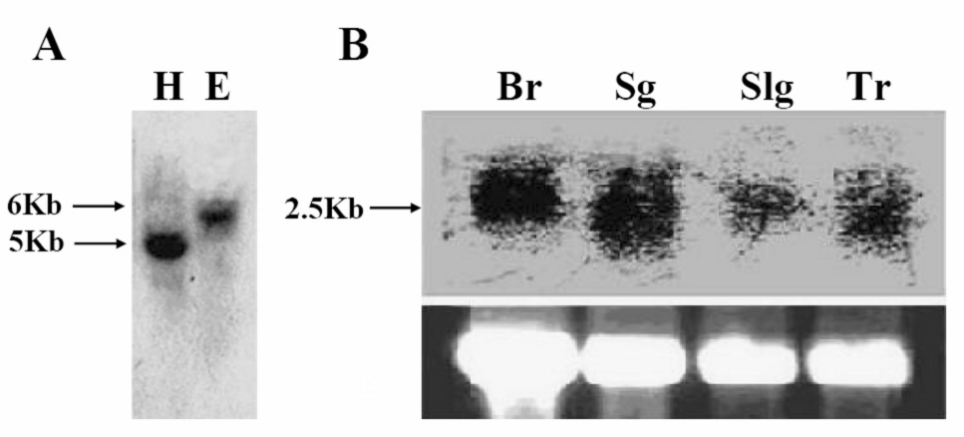
**Southern and northern blot analyses**. (A) Southern analysis. 10 μg of *H. armigera *genomic DNA was digested with *Hin*d III (lane H) and *Eco*R I (lane E), and hybridized with ^32^P-labeled Har-POU cDNA fragment. The sizes of the hybridizing fragments are indicated on the left. (B) Northern analysis. Total RNA (25 μg) was loaded on each lane, and the bottom panel shows the amount of ribosomal RNA loaded per lane (ethidium staining) as a control for loading variation. Br, brain; Sg, suboesophageal ganglion; Slg, salivary gland; Tr, trachea.

The copy number of the POU gene in the *H. armigera *genome was determined by Southern blotting. The cDNA fragment corresponding to the N-terminal and POU domain of Har-POU was used as a probe. As shown in Fig. 3A, only a single band of 5–6 kb was detected in the genomic DNA digested with *Hin*d III or *Eco*R I. Thus, Har-POU is probably encoded by a single gene in the *H. armigera *genome.

### Tissue distribution of Har-POU mRNA

Tissue distribution of Har-POU mRNA was detected by northern blot analysis. A band of about 2.5 kb was detected from the brain, SG, trachea, and salivary gland, indicating that the characterized cDNA cloned by RACE represents the full-length mRNA (Fig. 3B). The tissue distribution of Har-POU is consistent with that of *drifter*, which is mainly expressed in migrating neurons and tracheal cells in *Drosophila *[12].

### Nuclear localization of Har-POU

**Figure 4:**
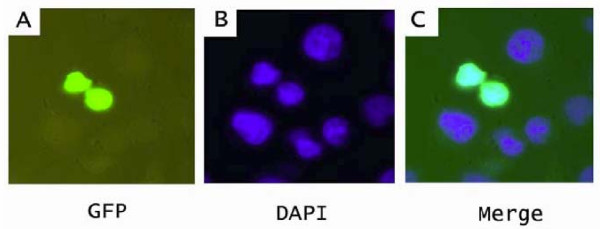
**Nuclear localization of Har-POU**. The eGFP-fused Har-POU was transfected into Hela cells. (A) The expression of Har-POU-eGFP; (B) DAPI staining to show the nuclei of the cells. (C) Overlapping of (A) and (B) to show that Har-POU-eGFP is located exclusively in the nuclei.

A basic sequence (GRKRKKRT) preceding helix 1 of the homeodomain was demonstrated to be a nuclear localization sequence in Oct-6, a member of the mammalian POU-III class [25]. This sequence is also conserved in invertebrate POU-III proteins including the Har-POU (Fig. 2A). To confirm whether Har-POU localizes to the nuclei efficiently, the eGFP-fused Har-POU was transfected into Hela cells. The eGFP-Har-POU exclusively localized to nuclei that were marked by DAPI (Fig. 4).

### DNA binding activities of Har-POU

**Figure 5:**
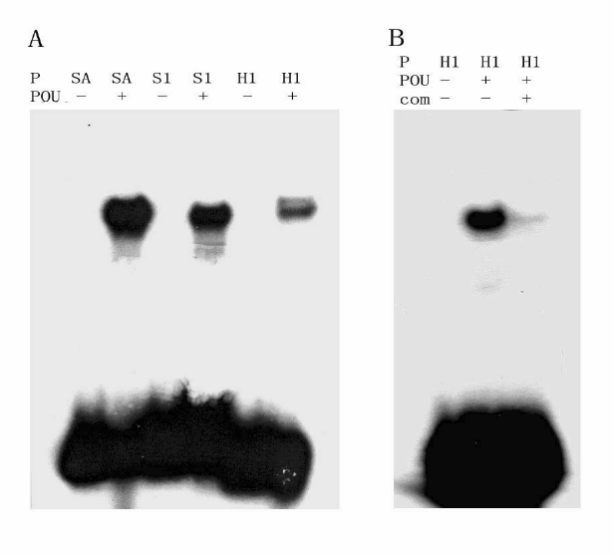
**Detection of Har-POU binding activity by electrophoresis mobility shift assay**. (A) Har-POU binds with probes SA, S1, and H1. (B) 40-fold of cold H1 specifically competes the biding of Har-POU and H1. The sequences of the probes SA, S1, and H1 are described in materials and methods. P, probe; POU, Har-POU protein expressed *in vitro*; com, cold H1 as a competitor.

Most of the POU proteins bind specifically to an octamer motif (ATGNAAAT). Three probes containing an octamer motif from insect genes were used in the EMSA assays: SA from the Bom-sericin promoter [26], S1 from the Bom-DH-PBAN promoter [5], and H1 from the Har-DH-PBAN promoter [21]. The *in vitro *translated Har-POU protein bound all three probes efficiently, and the binding could be competed by 40-fold unlabeled H1 (Fig. 5).

### Har-POU expression is related to pupal diapause

**Figure 6:**
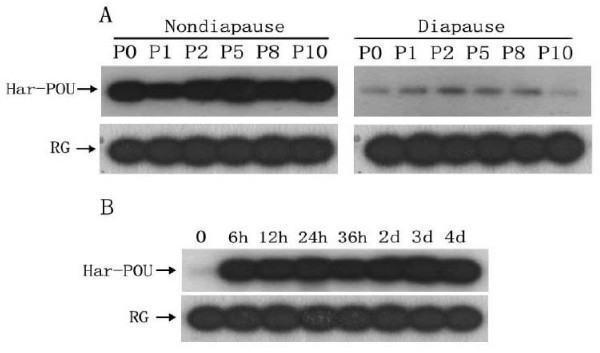
**Developmental expression of Har-POU mRNA detected by semi-quantitative RT-PCR and Southern hybridization**. (A) Expression of Har-POU mRNA during pupal development of nondiapause-destined and diapause-destined individuals. Abbreviations: P0–P10, 0–10 days after pupation; RG, rabbit globin. (B) Expression of Har-POU mRNA in diapausing pupae. Abbreviations h and d represent hours and days after injection of ecdysone to diapausing pupa.

Previous studies have demonstrated that expression of the Har-DH-PBAN gene is high in nondiapause-destined pupal individuals, but low in diapause-destined ones. Since we showed that Har-POU bound to the Har-DH-PBAN promoter, we investigated the developmental patterns of Har-POU mRNA in the two types of pupal individuals using RT-PCR combined with Southern blot. Har-POU mRNA content in nondiapause-destined individuals was robust from day 0 to day 10, whereas the Har-POU mRNA level of diapause-destined individuals at the corresponding time points was constantly low (Fig. 6A). The results show a close relationship between the gene expression of Har-POU and Har-DH-PBAN. Further, we investigated the developmental changes in Har-POU mRNA when pupal diapause was broken by injection of ecdysone. Har-POU mRNA in diapausing pupae increased to a high level 6 hours after injection and remained at consistently high levels from pupal development to the adult stage (Fig. 6B).

## Discussion and Conclusion

The studies presented here show: 1) cloning and characterization of Har-POU, a POU-III class gene in *H. armigera*; 2) that only a single copy of Har-POU exists in the *H. armigera *haploid genome; 3) Har-POU is expressed in various tissues and highly expressed in nervous, tracheal, and secretory tissues; 4) Har-POU localizes to the nuclei and binds three different probes containing octamer motifs; 5) Har-POU mRNA is highly expressed in developing individuals (nondiapause-destined pupae) and expressed at low levels in the programmed development-arrest individuals (diapause-destined pupae); 6) when pupal diapause is broken by injection of ecdysone, Har-POU gene expression immediately responds to restart development. These data imply that Har-POU's structure and function are conserved with POU-M1/-M2 in *B. mori *and *drifter *in *D. melanogaster*.

Usually, there is only one POU-III class gene in each invertebrate species, including *D. melanogaster *[11], *C. elegans *[8], crustacean *A. franciscana *[24], and the urochordate *O. dioica *at the invertebrate-vertebrate transition [9]. From the complete genome of several species, it has been shown that there are four POU-III genes in Human and mouse, but only one gene in invertebrates, including *Drosophila *and *C. elegans *[8]. Our results also show that there is only one copy of Har-POU in *H. armigera*. Interestingly, there are two isoforms of POU-III class genes in *B. mori*: POU-M1 and -M2, which were cloned in Japanese and Chinese *B. mori*, respectively [5,20]. These two proteins differ by a 28-amino acid sequence at their N-terminal (from residue 106 to residue 133) (Fig. 2B). This difference is produced by the insertion and loss of nucleotide acids, possible evidence of a polymorphism in *B. mori *strains. Since both *B. mori *and *H. armigera *belong to Lepidoptera, the similarity of the protein sequences can tell us which one is the main isoform in *B. mori*. As shown in Fig. 2B, 25 of the 28 amino acids of Har-POU are the same as that of POU-M2, and only 4 amino acids are conserved between Har-POU and POU-M1. Thus, we propose that POU-M2 may be the main isoform in Lepidoptera.

Diapause insects provide a good model for studying development and metabolism. *H. armigera *is a pupal diapause species, and its nervous system development stops after pupation. *drifter *in *Drosophila *has multiple functions in neuronal migration, lineage, and wiring [12,16-19]. Our results show that Har-POU expression is robust in the CNS of nondiapause pupae but very low in diapause-destined ones, suggesting that Har-POU has important functions in the development and remodeling of the *H. armigera *nervous system. The expression pattern of Har-POU is almost identical to that of Har-DH-PBAN. It is known that DH terminates diapause and promotes continuous development in *Heliothis virescens, H. armigera*, and *Helicoverpa assulta *[23,27,28]. Therefore, we propose that Har-POU might play a key role in regulating development by changing the Har-DH-PBAN transcript. In addition, Har-POU responds to the injection of ecdysone, well-known to regulate genes related to insect development [29-31]; thus it will be interesting to know whether Har-POU is its direct target.

## Methods

### Animals

*H. armigera *larvae were reared on an artificial diet at 22–23°C, 60% relative humidity, under a photoperiod of L14:D10 (light:dark) to produce nondiapause pupae, and at 20–21°C with a cycle of L10:D14 to induce diapause-type pupae.

To break diapause, 5 μl of a solution containing 1 μg 20-hydroxyecdysone (Sigma) or 5 μl distilled water as control was injected into *H. armigera *diapausing pupae through a fine glass capillary. The trachea, salivary gland from 6^th ^instar larval stage, and brain, SG or brain-SG complex from pupal stage, were dissected in insect saline containing 0.75% NaCl and stored at -70°C for RNA extraction.

### Cloning and sequence analysis

Total RNA was prepared from the brain-SG complexes of day-8 pupae (pharate adult). RNA extraction, RNA quantification, and reverse transcription were the same as described previously [23]. Two degenerate primers POUF (5'-CCATGGCGGCGAC(C/G)AC(C/G)TA(C/T)ATG-3') and POUR (5'-CAGCGTGTTGGG(C/T)GG(C/T)GTCAT-3') designed based on the highly conserved regions of *B. mori *POU-M2 and *D. melanogaster drifter *were used for PCR amplification under the following conditions: three cycles of 94°C, 45 s; 45°C, 1 min; 72°C, 45 s, and then 30 cycles of 94°C, 30 s; 50°C, 1 min; 72°C, 45 s.

To obtain the full-length Har-POU cDNA, rapid amplification of cDNA ends (RACE) was used. Specific primers (POU5-1 and POU5-2 for 5'-RACE, and POU3-1 and POU3-2 for 3'-RACE) were designed according to the sequence obtained from the internal amplification above. RACE reactions were performed with a SMART™ RACE kit (Clontech) according to standard protocol.

The PCR products were separated on a 1.2% agarose gel and ligated into a T-vector (TaKaRa). The recombinant DNA was transformed into *E. coli*, XL1-Blue competent cells. Positive clones were selected, and the isolated recombinant DNA was sequenced by TaKaRa Co. (Dalian, China).

### Semi-quantitative RT-PCR

Total RNA was prepared from the brain-SG of pupae. Total RNA from one brain-SG (about 1.5 μg) was reverse-transcribed. To normalize the efficiency of RNA reverse transcription in each reaction, 0.1 ng of rabbit globin (RG) mRNA (Promega) was added as external standard [22]. The primers and programs for PCR were the same as above, except the number of cycles was decreased from 30 to 20. The PCR products were electrophoresed on a 1.2% agarose gel and transferred onto a Hybond N^+ ^Nylon membrane (Amersham). The Har-POU cDNA was labeled with [α-^32^P]-dCTP as a probe using a random primed DNA labeling kit (TaKaRa). Nylon membrane was prehybridized for 4 h followed by addition of the radiolabeled probe for 18 h at 42°C in 5× SSPE (1× SSPE = 180 mM NaCl, 10 mM sodium phosphate, pH 7.7, 1 mM EDTA) containing 50% formamide, 5× Denhardt's solution, 0.1% SDS, and 100 μg/ml salmon sperm DNA. After hybridization, the membrane was washed with 0.2× SSPE at 45°C and finally exposed to the X-ray film for 20 h at -70°C.

### Northern and Southern blot analyses

For northern analysis, 25 μg of total RNA was separated on a 1.2% agarose gel containing 0.22 M formaldehyde and ethidium bromide, and subsequently blotted onto Hybond N^+ ^membrane.

Genomic DNA was isolated from the adult body of *H. armigera* using the procedure of Xu *et al*. [4]. 10 μg of high-molecular-weight genomic DNA was prepared, digested with restriction endonucleases, electrophoresed on a 1.0% agarose gel, and transferred to Hybond N^+ ^membrane. The hybridization probe and conditions and signal detection were the same as above.

### Construction of expression system

The open reading frame of Har-POU was amplified by the primers POU-EF (5'-CGGGATCCCCATGGCGGCGACCACGTACATG-3') and POU-ER (5'-CCCAAGCTTTTAGTGCGCGGCCAGCGTGTGC-3'). The underlined sequences correspond to *Bam*H I and *Hin*d III restriction sites. The PCR products were purified, digested by *Bam*H I and *Hin*d III, and cloned into pBluescript KS (+) or pEGFP-C1 (Clontech) plasmid. The recombinant plasmids were named T7-Har-POU and eGFP-Har-POU.

### In vitro translation and electrophoresis mobility shift assay (EMSA)

T7-POU plasmid DNA (1 μg) was used as a template for *in vitro *translation in the TNT Quick Coupled Transcription/Translation System (Promega) containing 40 μl of TNT T7 Quick Master Mix, 1 μl of methionine (1 mM), and 8 μl of distilled water. The reaction was allowed to proceed at 30°C for 1.5 h, and 2 μl of translation product was then used for the EMSA assay.

The probes used in EMSA were SA (5'-CTTGTATACATTGTTTGCAC AAATGTTTG-3') at -81 to -109 of Bom-sericin promoter [25], S1 (5'-CCCCTCATTTACATACATCCCCGTCCGAC-3') at -80 to -52 of the Bom-DH-PBAN promoter [5], and H1 (5'-TCCCTGATTTACATAAGAT TTCCATTCG-3') at -64 to -37 of the Har-DH-PBAN promoter [21].

In general, 10 fmol of ^32^P-labeled probe was incubated with 2 μl of translated product for 30 min at 27°C in a 20 μl reaction mixture containing 10 mM HEPES-K^+ ^(pH 7.9), 10% glycerol, 50 mM KCl, 4 mM MgCl_2_, 1 mM DTT, 0.5 mg/ml BSA, 0.1 mM PMSF and 1 μg of poly (dI-dC) (Pharmacia). Reaction mixtures were loaded onto a 5% native polyacrylamide gel and electrophoresed in 1× TBE buffer. After electrophoresis, the gel was dried and subjected to autoradiography in the presence of an intensifying screen at -70°C for 16 h. Competition assay was performed by preincubating the reactions with the specified amount of excess unlabeled probes for 10 min before the addition of labeled probes.

### Intracellular localization assay

Human cervical cancer cell line Hela was cultured in Dulbecco's modified Eagle's medium supplemented with 10% fetal bovine serum (FBS) and penicillin (100 μ/ml)/streptomycin (0.1 mg/ml) at 37°C in 5% CO_2_. Transfections of cells were performed using Lipofectamine 2000 (Invitrogen) according to manufacturer's instructions. Each co-transfection was performed in duplicate. The cell nuclei were counter-stained with DAPI and visualized with an inverse fluorescence microscope (Olympus IX70).

## Authors' contributions

TZ carried out all of experiments and wrote the manuscript. WX conceived the project, supervised the experiments and co-wrote the manuscript. All authors read and approved the final manuscript.
